# Maternal dietary patterns during pregnancy and intelligence quotients in the offspring at 8 years of age: Findings from the ALSPAC cohort

**DOI:** 10.1111/mcn.12431

**Published:** 2017-03-02

**Authors:** Ana Amélia Freitas‐Vilela, Rebecca M. Pearson, Pauline Emmett, Jon Heron, Andrew D. A. C. Smith, Alan Emond, Joseph R. Hibbeln, Maria Beatriz Trindade Castro, Gilberto Kac

**Affiliations:** ^1^ Nutritional Epidemiology Observatory, Department of Social and Applied Nutrition, Institute of Nutrition Josué de Castro Rio de Janeiro Federal University Rio de Janeiro Brazil; ^2^ School of Social and Community Medicine University of Bristol Bristol UK; ^3^ Section of Nutritional Neurosciences, Laboratory of Membrane Biology and Biophysics National Institute on Alcohol Abuse and Alcoholism, National Institutes of Health Bethesda Maryland USA

**Keywords:** ALSPAC, children, cluster analysis, dietary patterns, intelligence quotient, pregnancy

## Abstract

Dietary intake during pregnancy may influence child neurodevelopment and cognitive function. This study aims to investigate the associations between dietary patterns obtained in pregnancy and intelligence quotients (IQ) among offspring at 8 years of age. Pregnant women enrolled in the Avon Longitudinal Study of Parents and Children completed a food frequency questionnaire at 32 weeks' gestation (*n* = 12,195). Dietary patterns were obtained by cluster analysis. Three clusters best described women's diets during pregnancy: “fruit and vegetables,” “meat and potatoes,” and “white bread and coffee.” The offspring's IQ at 8 years of age was assessed using the Wechsler Intelligence Scale for Children. Models, using variables correlated to IQ data, were performed to impute missing values. Linear regression models were employed to investigate associations between the maternal clusters and IQ in childhood. Children of women who were classified in the meat and potatoes cluster and white bread and coffee cluster during pregnancy had lower average verbal (β = −1.74; *p* < .001 and β = −3.05; *p* < .001), performance (β = −1.26; *p* = .011 and β = −1.75; *p* < .001), and full‐scale IQ (β = −1.74; *p* < .001 and β = −2.79; *p* < .001) at 8 years of age when compared to children of mothers in the fruit and vegetables cluster in imputed models of IQ and all confounders, after adjustment for a wide range of known confounders including maternal education. The pregnant women who were classified in the fruit and vegetables cluster had offspring with higher average IQ compared with offspring of mothers in the meat and potatoes cluster and white bread and coffee cluster.

## 1 INTRODUCTION

General intellectual functioning is described by the intelligence quotients (IQ), which refers to general cognitive capacity, such as learning ability, reasoning, and problem solving (DSM IV, 1994). The first stage of brain development begins 18 days after fertilisation and continues long after birth; however, the brain's fastest growth occurs in utero, a vulnerable and critical period. Suboptimal nutrition during brain development may affect cognitive development and behavioural performance over time (Anjos et al., 2013; Rees & Inder, 2005; Thompson & Nelson, 2001).

During pregnancy, important neurologic functions are developing in the fetus (Rees & Inder, 2005). Brain development in the last trimester of gestation is particularly vulnerable to inadequacy in the mother's diet (Anjos et al., 2013). Specific aspects of maternal diet have long‐term positive associations with offspring neurodevelopment, including cognitive, psychomotor and mental development, IQ scores (verbal, verbal‐executive function, and performance), effects on behavioural status, and others (Anjos et al., 2013; Hibbeln et al., 2007; Gil & Gil, 2015; Starling, Charlton, McMahon, & Lucas, 2015). Intakes of specific food items, such as fish, during pregnancy have shown positive associations with neurodevelopmental outcomes in childhood (Anjos et al., 2013; Gil & Gil, 2015; Starling et al., 2015).

The study of isolated nutrients or food groups is helpful but does not fully capture the impact of nutrient interactions and the net effect of inadequate nutrient intakes in complex combinations. The derivation of dietary patterns is considered an appropriate way to assess dietary intake, as this method allows the evaluation of a combination of different types of foods consumed simultaneously. They can summarise the usual dietary intake of population groups facilitating the assessment of the overall diet effect on particular outcomes (Hu, 2002; Newby & Tucker, 2004). Principal component analysis (PCA) and cluster analysis have both been used to assess diet. Although in PCA, all subjects are included in all dietary patterns, creating food groupings based on correlations of dietary intake; in cluster analysis, individuals are classified into mutually exclusive and nonoverlapping clusters of subjects who consume similar foods (Bailey et al., 2006; Devlin, McNulty, Nugent, & Gibney, 2012; Hu, 2002; Newby & Tucker, 2004; Smith, Emmett, Newby, & Northstone, 2011; Wirfält, Drake, & Wallström, 2013). The ability of cluster analysis to aggregate subjects into exclusive groups aids interpretation of the relationship between the pattern and the outcome of interest (Devlin et al., 2012; Newby & Tucker, 2004) and is particularly helpful in longitudinal analysis. Other methods of assessing whole diet such as reduced rank regression and predefined dietary scores require prior reasonably robust evidence of the relationship between diet and the outcome being studied, which is not available in this case (Hoffmann, Schulze, Schienkiewitz, Nothlings, & Boeing, 2004).

Despite its ability to add insight into the relationship between diet and pregnancy outcomes, there are currently very few studies, which have used cluster analysis to derive dietary patterns during pregnancy (Vilela et al*.*, 2016). Therefore, this study will use it to obtain dietary patterns in pregnancy in the Avon Longitudinal Study of Parents and Children (ALSPAC), which has not been done before (Emmett, Jones, & Northstone, 2015).

Studies have examined cross‐sectional associations between dietary patterns and cognitive outcomes, in childhood, in adolescence, and in the elderly (Gale et al., 2009; Kim et al., 2015; Leventakou et al., 2016; Northstone, Joinson, Emmett, Ness, & Paus, 2012; Nyaradi et al., 2014). These studies have generally shown that higher scores on dietary patterns characterised by healthy foods (such as fruits, vegetables, and fish), measured in these stages of life, are associated with better cognitive outcomes, including higher childhood IQ. In addition, higher scores on unhealthy dietary patterns are generally associated with poorer cognitive outcomes in childhood and adolescence (Gale et al., 2009; Kim et al., 2015; Leventakou et al., 2016; Northstone et al., 2012; Nyaradi et al., 2014; Smithers et al., 2012; Smithers et al., 2013).

These studies highlight the importance of dietary intake at several stages of life. Maternal dietary intakes are clearly a dominant determinant of fetal nutrition in utero. However, the effects of maternal dietary patterns, obtained by cluster analysis or PCA, during pregnancy on neurodevelopmental outcomes in childhood are unknown. Therefore, the purpose of this study was to investigate the associations between maternal dietary patterns obtained by cluster analysis during pregnancy and IQ evaluated among offspring at 8 years of age.

**Key messages**

- The children of women in “fruit and vegetables” cluster had the highest mean verbal, performance, and full‐scale IQ scores in childhood compared to children with mothers classified in the “meat and potatoes” and “white bread and coffee” clusters during pregnancy, and children of women in white bread and coffee had the lowest average scores.
- In the current study, controlling for child's cluster pattern at 7 years of age did not remove the association with maternal diet in pregnancy, suggesting childhood diet did not completely explain the observed associations.
- Imputation of missing data did not change the associations between maternal dietary patterns and IQ at 8 years of age.

## 2 METHODS

### 2.1 Sample

**Figure 1:**
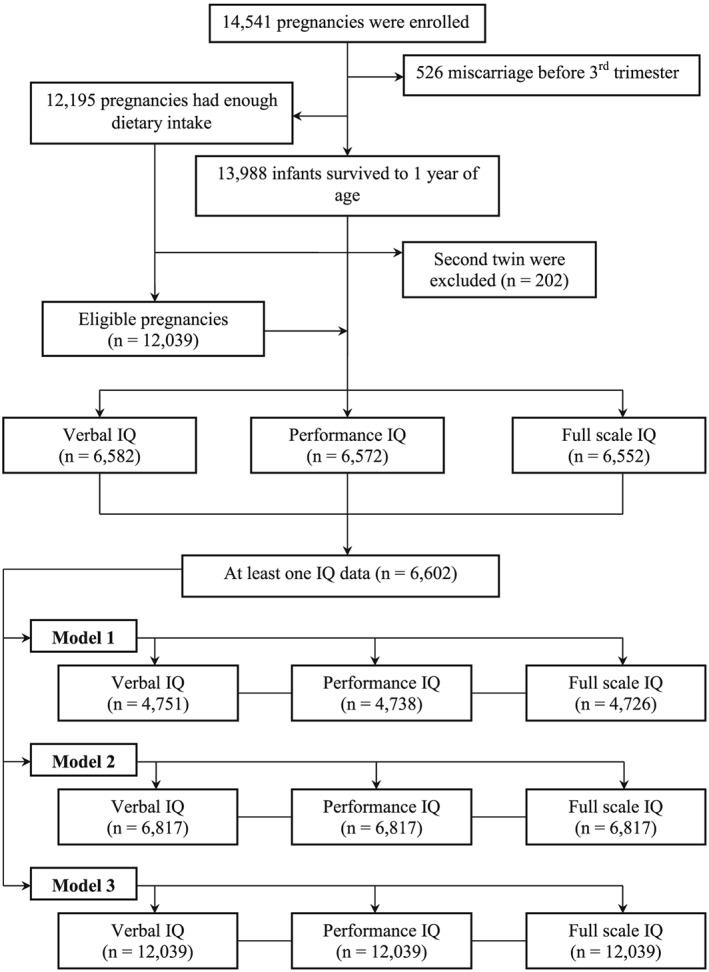
Flow chart illustrating the participant data of the study. Model 1, all available data; Model 2, imputations for missing data of intelligence quotients (IQ) and all confounders, only up to the sample for complete the IQ scale at 8 years and child neurodevelopment data assessed before 8 years of age, which were correlated to IQ; Model 3, imputations for missing data of IQ and all confounders

ALSPAC is a prospective cohort of pregnant women and their partners and offspring residing in the former county of Avon in Southwest England. It was designed to investigate the development of health and disease during pregnancy, childhood, and beyond (Boyd et al., 2013; Golding et al., 2001). Pregnant women who had an estimated date of delivery between April 1, 1991, and December 31, 1992, were eligible and invited for this study. A cohort of 14,541 pregnancies was established, and 13,988 infants survived to 1 year of age. Ethical approval for the study was obtained by ALSPAC Law and Ethics Committee and Local Research Ethics Committees. Details of all available data can be found through a fully searchable data dictionary (http://www.bris.ac.uk/alspac/researchers/data-access/data-dictionary). More information about ALSPAC is available at website (http://www.bristol.ac.uk/alspac/). In this study, we included singleton and first‐twins births as proposed in previous study from ALSPAC (Hibbeln et al., 2007). Figure 1 presents a flow chart for this cohort showing the number of subjects included in each step of this study.

### 2.2 Maternal dietary patterns

A total of 47 food items were used to obtain the clusters. All the dietary data were standardised by subtracting the mean and dividing by the range for each variable. The analyses were performed for two to seven clusters. The amount of variation explained by the solution, the size and interpretation of each cluster, and the stability of the solution, which was evaluated using linear discriminant analysis, were the criteria to choose the best cluster solution.

Three maternal dietary patterns during pregnancy were obtained by *k*‐means clustering. The complete cluster derivation methods were described in a previous publication by Vilela et al. (2016). The *k*‐means method derives clusters based upon the mean intakes of the input variables, using the squared Euclidian distances between observations to determine cluster position (Newby & Tucker, 2004). The fruit and vegetables cluster (*n* = 4,478) women had the highest frequency of consumption of nonwhite bread, fish, cheese, pulses, nuts, pasta, rice, vegetables, salad, fruit, and fruit juice when compared to the other clusters. The meat and potatoes cluster (*n* = 2,469) women had the highest frequency of consumption of all types of potatoes, red meat, meat pies, sausages and burgers, pizza, baked beans, peas, and fried foods compared to the other clusters. In the largest cluster, white bread and coffee (*n* = 5,248), the most characteristic foods were white bread, coffee, cola, and full‐fat milk; although in contrast, many of the foods associated with the other two clusters were consumed less frequently, especially those that defined the fruit and vegetables cluster.

### 2.3 Intelligence quotients

At 8 years of age, all children enrolled in ALSPAC were invited to attend a research clinic where trained psychologists measured their IQ using an adapted form of the Wechsler Intelligence Scale for Children‐III (Wechsler, Golombok, & Rust, 1992). The raw scores were age adjusted to determine verbal, performance, and full‐scale IQ (Joinson, Heron, Butler, Emond, & Golding, 2007).

### 2.4 Confounding variables

We selected variables that were known to be associated with diet and neurodevelopmental outcomes in childhood (Hibbeln et al., 2007). The maternal and child characteristics were obtained by self‐completed postal questionnaires answered by the mother at 8, 18, and 32 weeks' gestation and 6 months postpartum. Confounding variables included maternal education, housing, crowding at home, partner present, maternal age, maternal smoking in pregnancy, maternal alcohol use in pregnancy, parity, ethnic origin, prepregnancy body mass index (BMI), child's sex, and age at IQ measurement. Maternal education was classified as low (no academic examinations or a vocational level training), medium (O level—academic examination usually taken at age 16 years) and high (A level—academic examination usually taken at age 18 years or degree). Prepregnancy BMI [weight (kg)/height (m)^2^] was calculated from the self‐reported weight and height at 12 weeks gestation. Breastfeeding, child's energy intake, and dietary cluster at 7 years—plant‐based, traditional British, and processed (Smith et al., 2011)—were also adjusted for in the analysis because they may directly influence neurodevelopment and be associated with maternal diet.

### 2.5 Statistical analysis

#### 2.5.1 Descriptive analysis

The confounder variables included in this study were compared between children with and without IQ data using Student's *t* test and the chi‐square test for continuous and categorical variables, respectively. Children with IQ data were included only if mothers' dietary pattern during pregnancy was available. Moreover, children without IQ data were those who did not attend the study follow‐up for IQ to be measured at 8 years of age although their mothers provided dietary intake data during pregnancy. The analysis of variance, the Tukey–Kramer method, and chi‐square test were applied to test the differences in confounding variable structures between maternal clusters. The Tukey–Kramer method was used to take into account all possible pairwise comparisons (Ludbrook, 1991).

#### 2.5.2 Multiple imputation and regression models

Considering the loss of follow‐up in longitudinal studies, multiple imputation can be used as an alternative to applying list‐wise deletion to missing data, which reduces statistical power and introduces biases if those with missing data show systematic differences to those with complete data. In ALSPAC, there is substantial information regarding the pattern of missing data and we used this to impute them. Multiple imputation by chained equations is a common method used to handle missing data (Sterne et al., 2009; White, Royston, & Wood, 2011). This method relies on the “missing at random” assumption where missing data are predictable from observed data. Therefore, the first step of imputation in this study was to verify the correlation between the IQ data and different neurodevelopment outcomes in childhood for which we had more complete data. Variables with correlations greater than 0.2 were used in the multiple imputation by chained equations models: vocabulary scores and grammar scores at 24 months, verbal and performance IQ at 49 months, fine motor at 42 months, and hyperactivity scores at 81 months (data not shown). The correlated variables of neurodevelopment outcomes were included in all models of imputation.

Two models of imputation were constructed for those with dietary intake data during pregnancy. In the first model, we imputed missing confounding variables data for the children with complete IQ measurements at 8 years and correlated neurodevelopment outcomes, which were listed above (*n* = 6,817). In the second model, the missing data of IQ and all confounders were imputed (*n* = 12,039). One hundred imputed datasets were generated and all variables were imputed simultaneously, using adequate multivariate imputation methods. The fraction of missing information (FMI) was used to assess if the number of imputations was sufficient for the analysis. The rule of thumb suggests that the number of imputations (*m*) should be at least equal to the percentage of incomplete cases in the dataset, which can be assessed by the following equation: *m* ≥ 100 * FMI (White et al., 2011). The multiple imputation of the variance estimator is poor unless the number of imputations, *m*, is sufficiently large; however, the appropriate number of imputations is uncertain when the MI estimated coefficients approach normality and the variance estimator becomes well estimated (StataCorp, 2013).

Unadjusted and adjusted linear regression models were performed to evaluate the association between dietary patterns during pregnancy and IQ at 8 years of age. Verbal, performance, and full‐scale IQ were assessed in separate models. The fruit and vegetables cluster was designated as the reference group because this cluster was defined by foods considered to be healthy. The multiple linear regressions were adjusted for all confounding variables listed previously. The linear regression models were performed in three different models: (a) all available data without imputation; (b) imputations for missing data of IQ and all confounders, only up to the sample for complete IQ scale at 8 years or another correlated neurodevelopment outcomes; and (c) imputations for missing data of IQ and all confounders included all subjects with eligible dietary intake data. The number of observations from Model 2 is higher than Model 1, because Model 1 included only nonimputed data. In contrast, Model 2 was imputed for full‐scale IQ data from the greater numbers in which the dimensions of IQ had been measured.

All analyses were performed with the use of statistical software package Stata v13.1. Imputation was performed with mi impute command from Stata as follows:

mi set wide
mi register imputed all variables of the study and those variables used to impute the missing data
mi impute chained (regress) continuous variables (logit) categorical variables, augment savetrace (local disk) add (100) rseed (250510).

## 3 RESULTS

For children with IQ data, compared to those without, the mothers were more likely to have high educational attainment (43.3% vs. 25.7%), to be nonsmokers (55.8% vs. 43.6%), nulliparous (46.6% vs. 42.9%), and to have breastfed their children (81.9% vs. 67.1%). The women in the fruit and vegetables cluster were more likely than those in the other clusters to have high education, to be of older age (≥30 years), nonsmokers, and to have breastfed their children. Women in the white bread and coffee cluster were more likely to show less favourable socioeconomic indicators when compared to those in the other two clusters. The comparison between children with and without IQ data revealed differences in the proportions of almost all covariables, except prepregnancy BMI (Table 1).

**Table 1:** Maternal and child characteristics of offspring with and without IQ data at 8 years of age and according to maternal dietary clusters in pregnancy^a^

Confounders^b^	Without IQ data	With IQ data^c^		Fruit and vegetables	Meat and potatoes	White bread and coffee	
Continuous	Mean (*SD*)	Mean (*SD*)	*p* value^d^	Mean (*SD*)	Mean (*SD*)	Mean (*SD*)	*p* value^e^
Prepregnancy BMI	22.9 (4.0)	22.9 (3.7)	.329	22.4 (3.2)^g^	22.6 (3.5)^g^	23.7 (4.2)^h^	<.001
Child's diet at 7 years (kJ)	7,743 (1,930)	7,637 (1,776)	.010	7,610 (1,700)^g^	7,984 (2,282)^h^	7,461 (2,486)^i^	<.001
Child's age at 8 year clinic (months)	–	103.4 (3.3)	–	103.3 (3.1)^g^	103.3 (3.1)^g^	103.7 (3.7)^h^	<.001

Categorical	*n* (%)	*n* (%)	*p* value^f^	*n* (%)	*n* (%)	*n* (%)	*p* value^e^
Maternal education			<.001				<.001
Low	2,146 (39.9)	1,430 (21.7)		662 (15.0)^g^	719 (29.5)^h^	2,195 (42.8)^h^	
Middle	1,856 (34.5)	2,299 (34.9)		1,304 (29.6)^g^	961 (39.5)^h^	1,890 (36.9)^h^	
High	1,383 (25.7)	2,854 (43.3)		2,442 (55.4)^g^	754 (31.0)^h^	1,041 (20.3)^h^	
Housing			<.001				<.001
Mortgaged/owned	3,364 (65.0)	5,454 (84.2)		3,681 (85.0)^g^	1,856 (78.4)^h^	3,281 (66.1)^i^	
Public/social housing	988 (19.1)	504 (7.8)		206 (4.8)^g^	262 (11.1)^h^	1,024 (20.6)^i^	
Other	826 (15.9)	522 (8.1)		441 (10.2)^g^	248 (10.5)^h^	659 (13.3)^i^	
Crowding at home (person/room)			<.001				<.001
≤0.75	3,425 (67.5)	5,187 (80.9)		3,585 (83.8)^g^	1,769 (75.6)^h^	3,258 (66.9)^i^	
>0.75	1,650 (32.5)	1,226 (19.1)		691 (16.2)^g^	571 (24.4)^h^	1,614 (33.1)^i^	
Partner			<.001				<.001
No	219 (4.2)	104 (1.6)		82 (1.9)^g^	49 (2.1)^g^	192 (3.8)^h^	
Yes	5,011 (95.8)	6,431 (98.4)		4,279 (98.1)^g^	2,339 (97.9)^g^	4,824 (96.2)^h^	
Maternal age at birth of child (years)			<.001				<.001
<20	350 (6.5)	113 (1.7)		64 (1.4)^g^	98 (4.0)^h^	301 (5.8)^i^	
≥20 to <30	3,411 (62.9)	3,440 (52.1)		2,052 (46.5)^g^	1,477 (60.6)^h^	3,322 (64.3)^i^	
≥30	1,659 (30.6)	3,049 (46.2)		2,301 (52.1)^g^	864 (35.4)^h^	1,543 (29.9)^i^	
Maternal smoking in pregnancy			<.001				<.001
Nonsmoker	2,268 (43.6)	3,632 (55.8)		2,476 (57.0)^g^	1,258 (52.9)^h^	2,166 (43.5)^i^	
Stopped	1,564 (30.1)	1,994 (30.7)		1,455 (33.5)^g^	695 (29.2)^h^	1,408 (28.3)^i^	
Still smoking	1,370 (26.3)	877 (13.5)		414 (9.5)^g^	425 (17.9)^h^	1,408 (28.3)^i^	

Categorical	*n* (%)	*n* (%)	*p* value^f^	*n* (%)	*n* (%)	*n* (%)	*p* value^e^
Maternal alcohol use in pregnancy			<.001				<.001
Nondrinker	513 (9.9)	384 (5.9)		241 (5.5)^g^	177 (7.4)^h^	479 (9.6)^i^	
Stopped	1,909 (36.8)	2,485 (38.1)		1,715 (39.4)^g^	846 (35.5)^h^	1,833 (36.9)^i^	
Still drinking	2,769 (53.3)	3,648 (56.0)		2,396 (55.1)^g^	1,361 (57.1)^h^	2,660 (53.5)^i^	
Parity (number of deliveries)			<.001				<.001
0	2,207 (42.9)	3,003 (46.6)		2,216 (51.4)^g^	921 (39.1)^h^	2,073 (42.1)^i^	
1	1,795 (34.9)	2,313 (35.9)		1,383 (32.1)^g^	968 (41.1)^h^	1,757 (35.7)^i^	
≥2	1,146 (22.2)	1,133 (17.5)		716 (16.5)^g^	468 (19.9)^h^	1,095 (22.2)^i^	
Ethnic origin			<.001				<.001
White	5,138 (96.4)	6,443 (98.2)		4,252 (97.2)^g^	2,380 (98.6)^h^	4,949 (97.0)^g^	
Black	79 (1.5)	46 (0.7)		42 (1.0)^g^	12 (0.5)^h^	71 (1.4)^g^	
Asian	112 (2.1)	71 (1.1)		81 (1.8)^g^	22 (0.9)^h^	80 (1.6)^g^	
Breastfeeding			<.001				<.001
Yes	2,766 (67.1)	5,073 (81.9)		3,513 (87.7)^g^	1,550 (73.2)^h^	2,776 (66.2)^i^	
No	1,358 (32.9)	1,124 (18.1)		493 (12.3)^g^	569 (26.8)^h^	1,420 (33.8)^i^	
Sex			<.001				.717
Male	2,898 (53.5)	3,290 (49.8)		2,283 (51.7)^g^	1,245 (51.1)^g^	2,660 (51.5)^g^	
Female	2,522 (46.5)	3,311 (50.2)		2,133 (48.3)^g^	1,194 (48.9)^g^	2,506 (48.5)^g^	
Child dietary patterns			<.001				<.001
Plant based	469 (22.1)	1,482 (26.2)		1,123 (35.2)^g^	321 (19.5)^h^	507 (17.2)^i^	
Traditional British	500 (23.6)	1,407 (24.8)		735 (23.1)^g^	442 (26.8)^h^	730 (24.7)^i^	
Processed	1,149 (54.3)	2,779 (49.0)		1,328 (41.7)^g^	885 (53.7)^h^	1,715 (58.1)^i^	

*Note*. BMI = body mass index; *SD* = standard deviation.

a Data without imputation.

b The left portion presents the confounding variable data for mothers whose children did not have IQ data and the right portion the data for mothers of children with IQ data.

c At least one IQ data (verbal or performance).

d *p* values refer to Student's *t* test.

e *p* values refer to analysis of variance test.

f *p* values refer to chi‐square test.

^g, h, i^ Where superscripts differ, there is a difference among variables according to the dietary clusters (Tukey–Kramer method).

In unadjusted direct comparisons, children of women in fruit and vegetables cluster had the highest mean verbal, performance, and full‐scale IQ scores in childhood compared to children with mothers in the other two clusters, and children of women in white bread and coffee had the lowest average scores (Table 2).

**Table 2:** IQ at 8 years of age (unadjusted, without imputation) according to maternal dietary clusters during pregnancy

		IQ data		Fruit and vegetables		Meat and potatoes		White bread and coffee	
	*n*	Mean (*SD*)	*n*	Mean (*SD*)	*n*	Mean (*SD*)	*n*	Mean (*SD*)	*p* value^a^
Verbal IQ	6,582	107.5 (16.7)	2,758	111.6 (16.7)^b^	1,385	106.6 (16.1)^c^	2,439	103.3 (16.0)^d^	<.001
Performance IQ	6,572	99.9 (17.1)	2,751	102.9 (16.8)^b^	1,384	99.1 (17.0)^c^	2,437	97.1 (16.9)^d^	<.001
Full‐scale IQ	6,552	104.5 (16.4)	2,746	108.6 (16.1)^b^	1,378	103.5 (16.2)^c^	2,428	100.5 (15.9)^d^	<.001

*Note*. *SD* = standard deviation.

a *p* values refer to analysis of variance test.

^b, c, d^ Where superscripts differ, there is a difference among variables according to the dietary clusters (Tukey–Kramer method).

In both the unadjusted and adjusted models, children of women classified in the meat and potatoes cluster and white bread and coffee cluster during pregnancy had lower mean IQ scores at 8 years of age when compared with children of women classified in the fruit and vegetables cluster, whether multiple imputations were applied or not (Table 3). The results observed in the associations between meat and potatoes cluster and white bread and coffee cluster and IQ in multiple imputations were verbal IQ: β = −1.74; 95% CI [−2.65, −0.83]; *p* < .001 and β = −3.05; 95% CI [−3.95, −2.15]; *p* < .001; performance IQ: β = −1.26; 95% CI [−2.23, −0.28]; *p* < .001 and β = −1.75; 95% CI [−2.70, −0.80]; *p* < .001; and full‐scale IQ: β = −1.74; 95% CI [−2.65, −0.83]; *p* < .001 and β = −2.79; 95% CI [−3.66, −1.92]; *p* < .001. Adjustment for the potential confounders greatly attenuated the associations in each case; for example, for full‐scale IQ in Model 1, attenuation was from 8.13 IQ points deficit unadjusted to 3.10 IQ points deficit adjusted between the fruit and vegetables cluster and the white bread and coffee cluster. Considering the largest FMI of the models, which range from 0.035 (verbal IQ: Model 1—unadjusted) to 0.790 (performance IQ: Model 3—adjusted), the 100 imputations were adequate for these analyses (Table 3).

**Table 3:** Unadjusted and adjusted models of linear regression between maternal dietary clusters during pregnancy and verbal, performance, and full scale of IQ at 8 years of age

	Verbal IQ				Performance IQ				Full‐scale IQ			
	*n*	β	95% CI	*p*	*n*	β	95% CI	*p*	*N*	β	95% CI	*p*
Model 1—unadjusted	6,582				6,572				6,552			
Fruit and vegetables		Reference				Reference				Reference		
Meat and potatoes		−5.00	−6.05, −3.95	<.001		−3.77	−4.86, −2.68	<.001		−5.08	−6.11, −4.04	<.001
White bread and coffee		−8.34	−9.23, −7.45	<.001		−5.75	−6.67, −4.83	<.001		−8.13	−9.00, −7.25	<.001
Model 1—adjusted^a^	4,751				4,738				4,726			
Fruit and vegetables		Reference				Reference				Reference		
Meat and potatoes		−1.92	−3.11, −0.72	.002		−1.92	−3.18, −0.66	.003		−2.25	−3.41, −1.09	<.001
White bread and coffee		−3.51	−4.61, −2.41	<.001		−1.76	−2.92, −0.59	.003		−3.10	−4.18, −2.03	<.001
Model 2—unadjusted^b^	6,817				6,817				6,817			
Fruit and vegetables		Reference				Reference				Reference		
Meat and potatoes		−4.97	−6.01, −3.92	<.001		−3.68	−4.77, −2.60	<.001		−4.97	−6.00, −3.93	<.001
White bread and coffee		−8.35	−9.23, −7.46	<.001		−5.74	−6.66, −4.81	<.001		−8.09	−8.96, −7.21	<.001
Model 2—adjusted^a^, ^c^	6,817				6,817				6,817			
Fruit and vegetables		Reference				Reference				Reference		
Meat and potatoes		−1.83	−2.84, −0.81	<.001		−1.46	−2.55, −0.38	.008		−1.90	−2.90, −0.90	<.001
White bread and coffee		−2.97	−3.89, −2.05	<.001		−1.62	−2.61, −0.63	.001		−2.67	−3.58, −1.76	<.001
Model 3—unadjusted^d^	12,039				12,039				12,039			
Fruit and vegetables		Reference				Reference				Reference		
Meat and potatoes		−5.46	−6.41, −4.52	<.001		−3.91	−4.89, −2.93	<.001		−5.38	−6.33, −4.43	<.001
White bread and coffee		−9.17	−10.03, −8.30	<.001		6.42	−7.29, −5.54	<.001		−8.94	−9.77, −8.10	<.001
Model 3—adjusted^a^, ^e^	12,039				12,039				12,039			
Fruit and vegetables		Reference				Reference				Reference		
Meat and potatoes		−1.74	−2.65, −0.83	<.001		−1.26	−2.23, −0.28	.011		−1.74	−2.65, −0.83	<.001
White bread and coffee		−3.05	−3.95, −2.15	<.001		−1.75	−2.70, −0.80	<.001		−2.79	−3.66, −1.92	<.001

*Note*.

Model 1, all available data; Model 2, imputations for missing data of IQ and all confounders, only up to the sample for complete the IQ scale at 8 years and child neurodevelopment data assessed before 8 years of age, which were correlated to IQ; Model 3, imputations for missing data of IQ and all confounders.

a All models were adjusted for maternal education, housing tenure, crowding at home, partner present, maternal age, maternal smoking in pregnancy, maternal alcohol use in pregnancy, parity, ethnic origin, prepregnancy BMI, breastfeeding, child sex, child's energy intake at 7 years of age, child's dietary patterns at 7 years of age, and child's age at IQ measurement.

Largest fraction of missing information:

b Verbal IQ = 0.035, performance IQ = 0.045, full‐scale IQ = 0.041;

c Verbal IQ = 0.175, performance IQ = 0.204, full‐scale IQ = 0.192;

d Verbal IQ = 0.413, performance IQ = 0.389, full‐scale IQ = 0.384;

e Verbal IQ = 0.735, performance IQ = 0.790, full‐scale IQ = 0.759.

## 4 DISCUSSION

The women who were classified in the meat and potatoes cluster and white bread and coffee cluster during pregnancy had children with lower mean verbal, performance, and full‐scale IQ at 8 years of age, when compared to children of women in the fruit and vegetables cluster in pregnancy. Adjusting for socioeconomic factors, breastfeeding and child diet attenuated the size of the association but did not remove the relationship completely. Applying imputation did not change the associations greatly. To our knowledge, this is the first study to investigate the association between maternal dietary clusters during pregnancy and offspring IQ in childhood.

### 4.1 Potential mechanisms

The children evaluated in this cohort presented higher mean IQ scores when compared to those from some other studies (Factor‐Litvak et al., 2014; Iglesias, Steenland, Maisonet, & Pino, 2011; Wasserman et al., 1997). This may be due to the relatively affluent circumstances of the women and children living in the UK at the turn of the 20th century where food is in abundant supply.

Individual nutrients provided by maternal diets during pregnancy have been investigated with respect to childhood neurological development. Nutrients such as iron and zinc have been associated with infant neurological development (Anjos et al., 2013; Gil & Gil, 2015; Starling et al., 2015). Adequate supplementation with folic acid during pregnancy has been associated with better neurological developmental in children under 18 months in two studies (Chatzi et al., 2012; Valera‐Gran et al., 2014). Investigations relating to particular foods have focused on fish, which is a good source of many nutrients, such as long‐chain polyunsaturated fatty acids, iodine, and many vitamins. In a previous study from ALSPAC, children of mothers who had lower intakes of fish or seafood during pregnancy were more likely to have suboptimum neurodevelopmental outcomes, including verbal and full‐scale IQ at 8 years of age (Hibbeln et al., 2007). Furthermore, in a subsample of pregnant ALSPAC women in early gestation, the urinary iodine concentrations were associated with child cognitive development; low maternal iodine status was associated with an increased risk of suboptimum scores for verbal IQ at 8 years of age (Bath, Steer, Golding, Emmett, & Rayman, 2013). In a mother–child cohort from North Eastern Italy, the intake of fresh fish during pregnancy showed a marginal positive association with full scale and performance IQ, but the intake of canned fish was negatively associated with verbal, performance, and full‐scale IQ at 7 years of age (Deroma et al., 2013). These results suggest that consuming a nutrient dense diet during pregnancy is important for an offspring's neurological development. This is supported by our previous study (Vilela et al., 2016), which found that women in the fruit and vegetables cluster had a diet that was more nutrient dense than that of women in the other two clusters. In the current study, we have shown that this better quality diet is associated with better neurological development in the offspring.

It is likely that mothers' diet will influence the diet of their children once they are born. In our previous study, we found this to be the case (Vilela et al., 2016); in particular, children of mothers in the fruit and vegetables cluster were more likely than those of mothers in the other two clusters to have their diets classified in a “plant‐based” cluster, which was characterised by very similar foods (Smith et al., 2011). There are a few other studies that have assessed dietary patterns, obtained by PCA, in childhood in relation to neurological outcomes (Gale et al., 2009; Leventakou et al., 2016; Northstone et al., 2012; Smithers et al., 2012; Smithers et al., 2013). Gale et al*.* (2009) obtain dietary patterns in 6 and 12 month old infants from the Southampton Women's Survey and the Wechsler Pre‐School and Primary Scale of Intelligence to measure the IQ and found an association between higher scores on an “infant guidelines” dietary pattern and higher full‐scale and verbal IQ at 4 years of age. Smithers et al. (2012) found that the high scores of dietary patterns in infancy, which included fruits, vegetables, and breastfeeding, were associated with higher mean IQ at 8 years of age. Furthermore, the high scores on dietary patterns, which included processed foods, were associated with lower mean average IQ at 8 years of age.

Dietary patterns in children at age 3 and 8 years showed an inverse association of a “processed” pattern and a positive association of a “health‐conscious” pattern, respectively, with IQ measured at 8 years of age, in ALSPAC Northstone et al. (2012). In Australia, Nyaradi et al. (2014) assessed adolescents from the Western Australian Pregnancy Cohort (Raine) Study and found that higher scores on a “western dietary pattern” at 14 years of age were associated with lower cognitive performance at 17 years of age. A study with older subjects from rural areas of South Korea identified two dietary patterns using the *k*‐means cluster analysis and found that subjects in a cluster composed by multigrain rice, fish, dairy products, and fruits and fruit juices presented lower cognitive impairment, when compared with those in a cluster constituted by white rice, noodles, and coffee. The cognitive function was measured by Mini‐Mental Status Examination‐Korean version (Kim et al., 2015). These results suggest that the dietary pattern of the person themselves is also influential in determining their cognitive status. In combination, these studies suggest that an alternative potential mechanism to the observed associations operating through in utero effects is that maternal diet during pregnancy predicts child diet once they are born and in turn child diet is associated with higher IQ. However, in the current study, controlling for child's cluster pattern at 7 years of age did not remove the association with maternal diet in pregnancy, suggesting childhood diet did not completely explain the observed associations.

### 4.2 Strengths and limitations

One limitation of this study is the loss to follow‐up of subjects from the original sample and missing data for some of the confounding variables, common problems in cohort studies. We used multiple imputations to try to account for this and found that the relationships were very similar with and without imputation. Another potential limitation refers to recall bias when completing a food frequency questionnaire, which may lead to underestimation or overestimation of the dietary intake. However, in this study, we asked women to recall their current diet over 1 month and used only the frequency of intake of each food, thus reducing recall bias as well as avoiding that due to inaccurate estimation of portion size. The food frequency questionnaire was designed to capture the intake of a UK population in 1990s; therefore, there may have been some changes to the types of diets consumed by pregnant women in the ensuing years. The cluster analyses were performed before the imputation models; it is likely that differences between clusters would be attenuated in this instance.

The strengths of this study include the large sample size, the long‐term follow‐up from pregnancy to childhood, the standardised instrument used to collect the IQ data, and the availability of a large number of prospectively collected confounding variables. The use of dietary patterns to summarise the diet rather than studying isolated nutrients or food intakes, which do not fully capture the complexity of the diet is a further strength. However, there is a possibility that some factors important for cognitive development have not been included in the analysis and that the associations are the result of residual confounding. It is certainly true that the associations were greatly attenuated in the fully adjusted models that included maternal education as a proxy for maternal intelligence.

In summary, the women whose food habits during pregnancy placed them in the meat and potatoes cluster and white bread and coffee cluster had children with lower mean IQ at 8 years of age, when compared to children of mothers whose food habits placed them in the fruit and vegetables cluster. These results add to previous findings that maternal diet in pregnancy is key to promoting optimal neurodevelopment in offspring and imply that support for good nutrition during pregnancy is likely to be cost effective. Further research particularly in less well‐nourished populations is needed to confirm and extend these findings.

## CONFLICTS OF INTEREST

The authors declare that they have no conflicts of interest.

## CONTRIBUTIONS

AAF‐V, PE, GK designed the research; PE, AE conducted the research; AAF‐V, PE, and GK wrote the paper; AAF‐V, RMP, ADACS analysed the data or performed statistical analysis; AAF‐V had primary responsibility for the final content. All authors have read and approved the final manuscript.
